# Supplementary material for: Optimization of Large Vessel Occlusion Detection in Acute Ischemic Stroke Using Machine Learning Methods
Source: Life (Basel). 2022 Feb 3;12(2):230. doi: 10.3390/life12020230 (PMC8877679; doi:10.3390/life12020230)
Supplement: Supplementary file 1 [file life-12-00230-s001.zip › life-1517036-supplementary.pdf]

Supplementary material

Optimization of large vessel occlusion detection in acute ischemic stroke using machine learning methods

Authors: Tarkanyi Gabor, Tenyi Akos, Hollos Roland, Kalmar Peter Janos, Szapary Laszlo

1. Study cohort

In this study we used data from the STAY ALIVE acute stroke registry, which is a part of the GINOP 2.3.2-15-2016-00048 Stay Alive project. This registry is a prospectively collected, ongoing, national, hospital-based, multicentre database of acute ischemic stroke patients including comprehensive stroke centres of three university hospitals in Hungary (University of Debrecen, University of Szeged, and University of Pécs). Patients who are admitted to one of these stroke centres due to acute ischemic stroke are prospectively screened and enrolled to the registry. Participation is voluntary and written informed consent is obtained from each patient. Detailed data on medical history, on admission parameters, imaging results, interventions, medical investigations, etiology and follow-up data are collected by clinical research administrators and medical doctors. Data are recorded on an electronic case report form (eCRF) and subsequently checked and approved by an assigned trained neurologist and by the chief research administrator. Final approval is made by the head of each department who are also the guarantors. Detailed information can be found at https://tm-centre.org/en/registries/stroke-registry/.

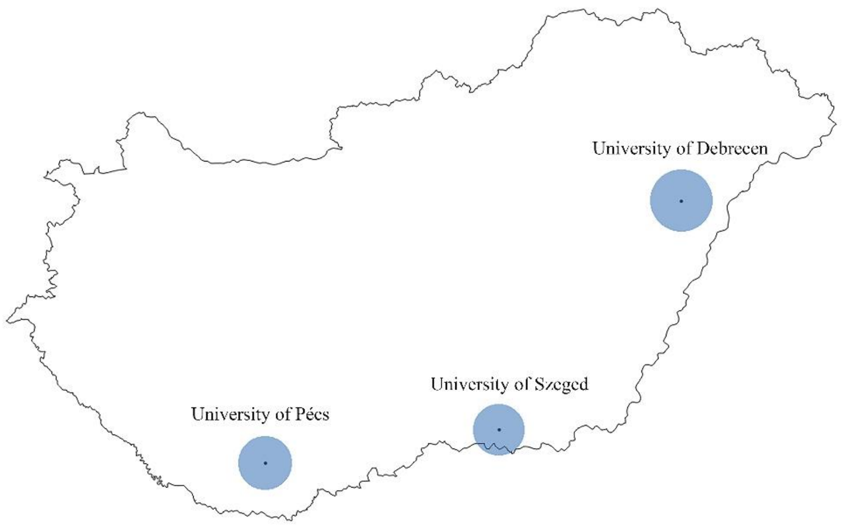

Institution	No. of screened patients	No. of patients underwent CTA	No. patients with LVO
University of Debrecen	240	202	78
University of Pécs	207	196	87
University of Szeged	199	128	62

Figure S1. Location of participating centres and distribution of patient enrolment.

Table S1. Proportions of missing values.

Variable	Category	All patients	Non LVO group	LVO group
Sex	BD	0,0%	0,0%	0,0%
Age	BD	0,0%	0,0%	0,0%
Onset-to-ER	X	0,0%	0,0%	0,0%

ER assessment-to-imaging	X	0,0%	0,0%	0,0%
Smoking	MH	12,5%	9,4%	16,7%
Hypertension	MH	2,7%	2,7%	2,6%
Diabetes	MH	3,8%	4,0%	3,5%
Chronic heart failure	MH	4,9%	5,7%	4,0%
Hyperlipidaemia	MH	7,4%	7,7%	7,0%
Previous stroke/TIA	MH	4,2%	4,7%	3,5%
Coronary artery disease	MH	6,1%	7,0%	4,8%
Atrial fibrillation	MH	4,2%	4,3%	4,0%
Malignancy	MH	6,3%	6,0%	6,6%
NIHSS	X	0,0%	0,0%	0,0%
SBP	BD	1,1%	1,0%	1,3%
DBP	BD	1,3%	1,3%	1,3%
BMI	BD	16,2%	13,0%	20,3%
SpO_2	X	42,0%	44,5%	38,8%
Body temperature	X	39,4%	35,1%	44,9%
Heart rate	BD	3,8%	3,7%	4,0%
Glucose	L	2,1%	1,3%	3,1%
Platelet	L	3,2%	2,7%	4,0%
Haematocrit	L	2,9%	2,3%	3,5%
Haemoglobin	X	2,9%	2,3%	3,5%
CRP	L	2,9%	2,7%	3,1%
Creatinine	L	2,1%	1,3%	3,1%
INR	L	4,9%	5,0%	4,8%
BUN	L	1,9%	1,0%	3,1%
AST	L	5,3%	5,0%	5,7%
ALT	L	5,3%	6,0%	4,4%
WBC	L	3,0%	2,3%	4,0%

Abbreviations: BD, baseline and demographic parameters; MH; medical history, L, laboratory value; ER, emergency room; TIA, transient ischemic attack; NIHSS, National Institutes of Health Stroke Scale; SBP, systolic blood pressure; DBP, diastolic blood pressure; BMI, body mass index; INR, international normalized ratio; BUN, blood urea nitrogen; AST, aspartate aminotransferase, ALT alanine aminotransferase; WBC, white blood cell.

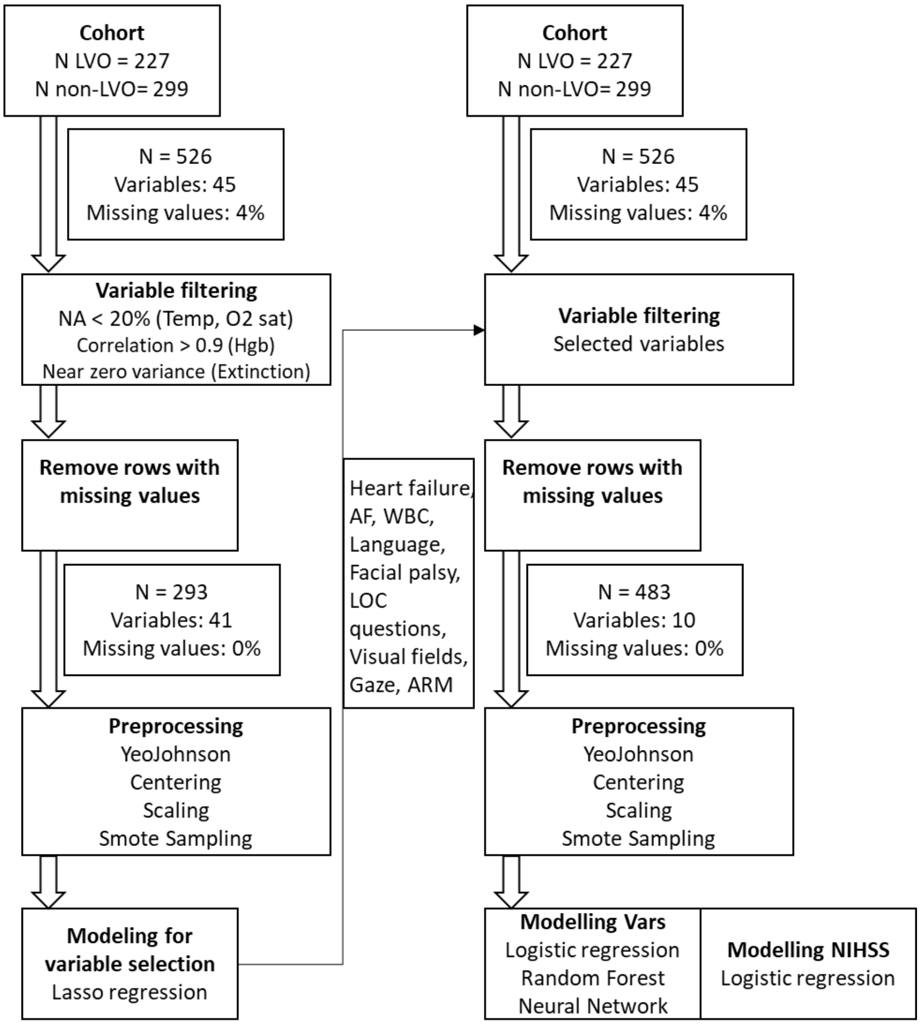

Figure S2. Chart of analysis workflow.

2. Missing value analysis and imputation

As the dataset contained missing values (see Table S1), we aimed at exploring potential biases introduced by missing values and their removal, as well as the effect of subsetting the dataset for the different analyses.

3. Missingness mechanisms

We compared the samples with missing values (n=233) vs. samples without missing values (n=293) to explore variables that are conditioned on missingness. Variables distributions of the two datasets were compare using Kolmogorov–Smirnov test (continuous variables) and Fisher exact test (binary variables).

Comparison showed two variables with significant difference (p-value < 0.05) between the subset with and without missing values (Figure S3).

- Dyslipidemia: missing values were more frequent in patients with dyslipidemia.
- Hospital: missing values were more frequent in certain hospitals than others. Proportion in samples with no missing values vs with missing values. - Hospital 1: 37% vs 43% - Hospital 2: 38% vs 25% - Hospital 3: 24% vs 31%

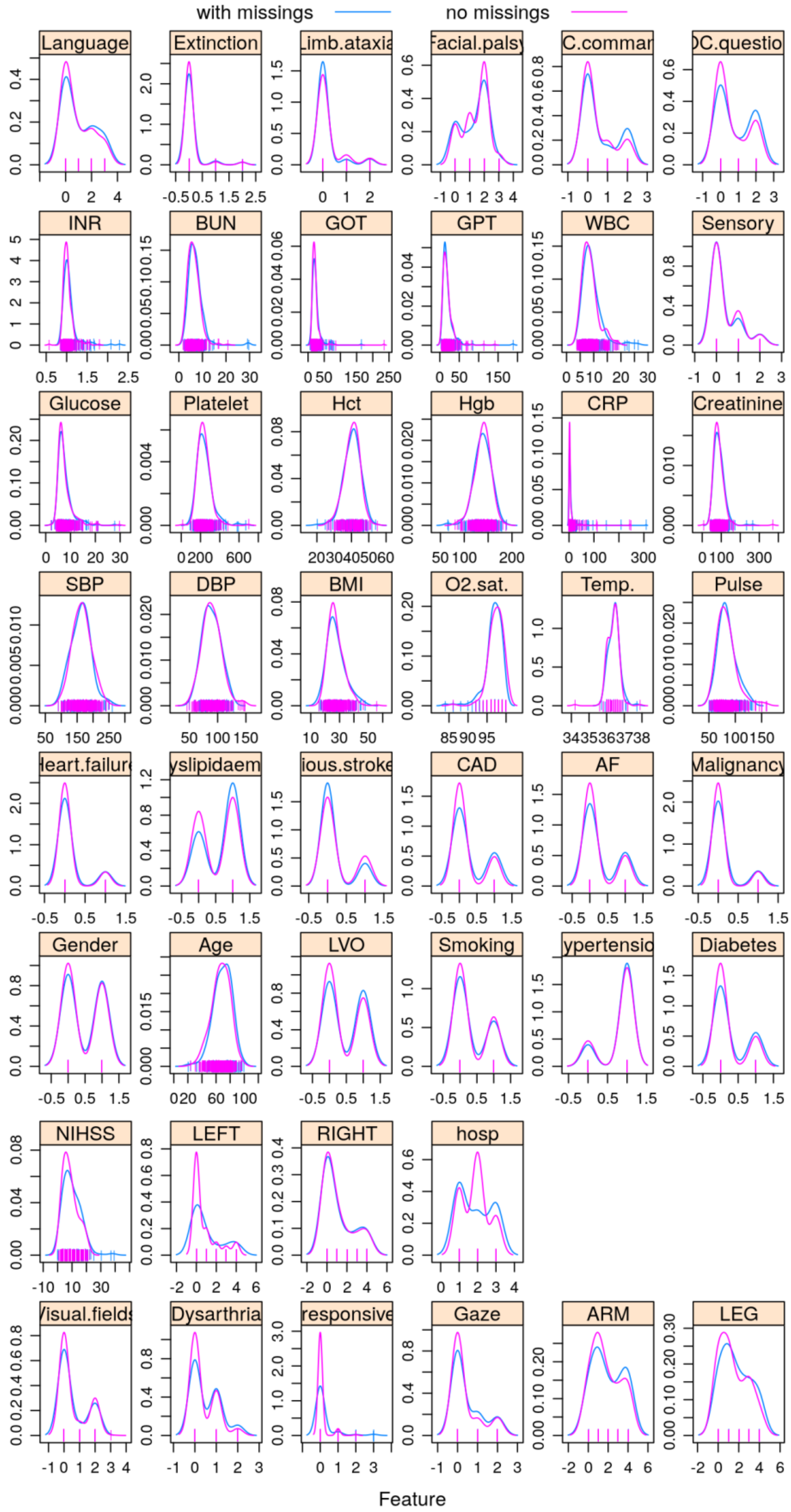

Figure S3. Comparison of the distribution of variables between samples with- and without missing values.

4. Effect of subsetting

4.1. Comparison of original dataset (n = 526) vs dataset used for variable selection (n=293)

The aim of this analysis is to explore the potential biases that omitting samples with missing variables would introduce. Variables distributions of the two datasets were compare using Kolmogorov–Smirnov test (continuous variables) and Fisher exact test (binary variables).

Variable filtering steps

- No data > 20% (Temp, O2 sat)
- Correlation > 0.9 (Hgb)
- Near zero variance (Extinction)
- No data omit

Results showed no significant differences between the datasets (Table S3). Preprocessed datasets should provide valid results representative to the initial population.

Table S2. Comparison of variables distributions of samples of the original dataset vs dataset used for variable selection. Only top 10 variable sorted by p-value is showed.

**Variable**	***P*-value**
Dyslipidaemia	0.233
Previous.stroke.TIA	0.340
CAD	0.344
LVO	0.377
Diabetes	0.393
AF	0.443
hosp	0.605
Hypertension	0.646
Age	0.694
Gender	0.715

4.2. Comparison of original dataset (n = 526) vs dataset used for model comparison after feature selection (n = 483)

The aim of this analysis is to explore the potential biases that preprocessing, including omitting samples with missing variables would introduce. Variables distributions of the two datasets were compare using Kolmogorov–Smirnov test (continuous variables) and Fisher exact test (binary variables).

Variable filtering steps

- Variable selection
- No data omit

Results showed no significant differences between the datasets. Preprocessed datasets should provide valid results representative to the initial population.

Table S3. Comparison of variables distributions of samples of the original dataset vs dataset used for model comparison after feature selection. Only top 10 variable sorted by p-value is showed.

**Variable**	**P-value**
Diabetes	0.658
Previous.stroke.TIA	0.761
Gender	0.800

CAD	0.824
Heart.failure	0.847
Malignancy	0.849
Hypertension	0.873
AF	0.942
Dyslipidaemia	0.947
LVO	0.949

5. Imputation method selection

To select the best missing value imputation strategy different imputation methods were evaluated and compared. In the initial dataset there was relatively high amount of missing data (4% of the dataset), which mainly showed missing at random properties (see Figure S3) and which was mainly concentrated in a few variables. Our analysis showed that imputing missing values would negatively affect the performance of the final models (see Figure S4), thus patients with missing values were omitted from the analysis and a two-step approach was followed to maximize sample size for modelling. Multiple imputation using Fully Conditional Specification (FCS) implemented by the MICE algorithm in R was used to test different imputation methodologies. The AUC of LVO prediction using logistic regression was used to compare the performance of the different imputation strategies.

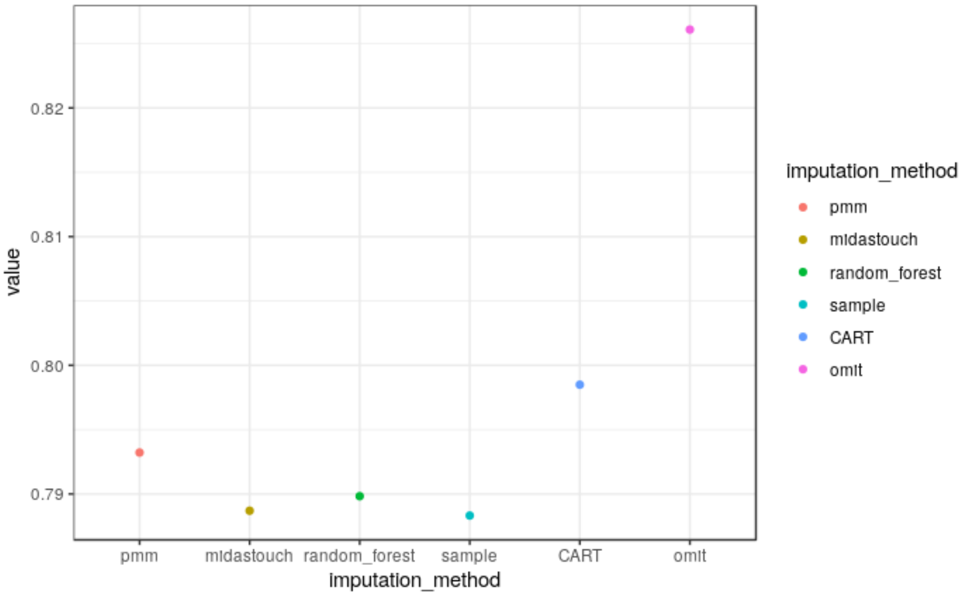

Figure S4. Effect of missing value imputation methods (predictive mean matching (PMM), midas touch, random forest, CART, random sampling, omitting missing values) on the performance of predicting LVO (measured by AUC) of the different imputation methodologies.
